# In Vivo Confocal Microscopy of the Corneal-Conjunctival Transition in the Evaluation of Epithelial Renewal after SLET

**DOI:** 10.3390/jcm9113574

**Published:** 2020-11-06

**Authors:** Emilio Pedrotti, Chiara Chierego, Tiziano Cozzini, Tommaso Merz, Neil Lagali, Alessandra De Gregorio, Adriano Fasolo, Erika Bonacci, Jacopo Bonetto, Giorgio Marchini

**Affiliations:** 1 Ophthalmic Unit, Department of Neurosciences, Biomedicine and Movement Sciences, University of Verona, Policlinico G.B. Rossi, P.le L.A. Scuro 10, 37134 Verona, Italy; emilio.pedrotti@univr.it (E.P.); chiara.chierego@aovr.veneto.it (C.C.); merz.tommaso@gmail.com (T.M.); adriano.fasolo@yahoo.it (A.F.); bonaccierika89@gmail.com (E.B.); jacopobonetto@libero.it (J.B.); giorgio.marchini@univr.it (G.M.); 2 Division of Ophthalmology, Department of Biomedical and Clinical Sciences, BKV, Linköping University, SE-581 83 Linköping, Sweden; neil.lagali@liu.se; 3 Ophthalmic Unit, San Bassiano Hospital, Via dei Lotti, 40, 36061 Bassano del Grappa, Italy; adegre3@gmail.com; 4 The Veneto Eye Bank Foundation, Padiglione G. Rama, Via Paccagnella 11, 30174 Zelarino Venezia, Italy

**Keywords:** in vivo confocal microscopy, simple limbal epithelial transplantation, corneal-conjunctival epithelial transition, keratoplasty, limbal stem cell deficiency

## Abstract

Examination of the corneal surface by in vivo confocal microscopy (IVCM) allows for objective identification of corneal and conjunctival cell phenotypes to evaluate different epithelialization patterns. Detection of a corneal-conjunctival epithelial transition could be considered as a sign of restored epithelial function following simple limbal epithelial transplantation (SLET). This is a prospective, interventional case series. We assessed patients with limbal stem cell deficiency (LSCD) by IVCM, preoperatively and at monthly intervals following SLET. Sectors in the central and peripheral cornea were scanned. Immediately upon detection of multi-layered cells with the epithelial phenotype in the central cornea and confirmation of epithelial transition in all corneal sectors, the decision for keratoplasty was taken. Ten patients were enrolled. After SLET, epithelial phenotype in the central cornea and an epithelial transition were identified within six and nine months in seven and one patients, respectively. One patient was a partial success and one failed. Five patients underwent keratoplasty, with stable results up to 12 months. Identification of the epithelial transition zone by IVCM permits assessment of the efficacy of SLET, enabling subsequent planning of keratoplasty for visual rehabilitation. The stability of the corneal surface following keratoplasty confirms that the renewal of the corneal epithelium was effectively retained.

## 1. Introduction

Limbal stem cell deficiency (LSCD) is a severe and debilitating condition affecting the ocular surface, caused by a decrease in the population and/or function of corneal epithelial stem cells located in the limbus. LSCD leads to eye discomfort, recurrent epithelial defects, mild to severe inflammation, stromal ulceration, and scarring [1]. Among the acquired causes of LSCD, ocular surface chemical or thermal burns are the most common causes, while other etiologies include chronic contact lens wear, multiple surgeries involving the limbus, infections, and toxic epidermal necrolysis [1].

In patients with total LSCD, the conjunctival epithelium invades the cornea over the entire 360-degree circumference, followed by sub-epithelial fibrosis, neovascularization, development of a fibrovascular pannus and loss of vision in advanced stages. In such cases, treatments aim to recover limbal function and may consist of a cell therapy procedure based on the transplantation of allogeneic limbal stem cells harvested from a donor cornea or autologous limbal stem cells from the patient’s healthy fellow eye. Techniques have evolved from conjunctival-limbal autografting (CLAU) to cultivated limbal stem cell transplantation (CLET). To overcome the drawbacks of the large biopsy of healthy tissue required for CLAU and the infrastructure and regulatory requirements for CLET, a technique of in situ limbal stem cell expansion, termed ‘simple limbal epithelial transplantation’ (SLET), has been developed [2,3,4,5]. A recent meta-analysis compared cultivated limbal transplant techniques and direct limbal transplantations (including SLET) and found more favorable outcomes for direct limbal transplantation [6].

Assessment of the actual recovery of limbal function and self-renewal of the epithelial cell population following any of the above-mentioned surgical techniques is crucial, because corneal grafting or other corrective or reconstructive procedures, often required in such patients, can safely and effectively be applied only after confirmation of recovered limbal function.

A hallmark of effective regeneration of the limbal stem cell function is the maintenance of a transparent and intact corneal epithelium, without central corneal superficial neovascularization, for a period of one year after surgery, which is consistent with a normal homeostasis of the entire human corneal epithelium estimated as ranging from nine to 12 months [7,8,9]. However, weighing the risk-benefit of further surgery after cell therapy for LSCD is a matter of concern, as approximately 50% of successful CLET procedures had shown subsequent corneal surface failure or recurrence of epithelial defects after corrective or reconstructive penetrating keratoplasty (PK) [10,11,12].

In vivo confocal microscopy (IVCM) provides a method for objective evaluation of corneal cellular structures at a pseudo-histological level. IVCM can be more accurate than biomicroscopy and more reliable than impression cytology in discriminating between different epithelialization patterns, recognizing corneal and conjunctival cell phenotype, and detecting the presence of other resident cells on the corneal surface [13,14]. The detection of the corneal-conjunctival epithelial transition, the transition from corneal to conjunctival cell morphology, is one of the most interesting aspects of IVCM examination in eyes with LSCD [15]. While absent in patients with total LSCD, such a watershed line recognized in the corneal periphery following surgery delineates a sort of novel limbus, and indicates achievement of the expected regeneration of the corneal epithelial surface [13].

In this study, we applied IVCM examination to diagnose LSCD and evaluate the clinical course after SLET, with the aim to assess the efficacy of the restoration of a stable characteristic renewing epithelium on the corneal surface by identifying the epithelial transition and to decide on subsequent keratoplasty (penetrating or deep anterior lamellar) in patients requiring it.

## 2. Experimental Section

### 2.1. Study Design and Patient Selection

This prospective interventional study was conducted at the Ophthalmic Unit of the University of Verona, between 2017 and 2019, following approval by the Review Board of the University of Verona (protocol no. 105878). We observed the tenets of the Declaration of Helsinki and all patients signed informed consent after a detailed explanation of the study.

We recruited consecutive patients aged 18 years and older diagnosed with total LSCD in one eye. We performed SLET to obtain surgical rehabilitation of limbal function and re-establish the proper corneal surface conditions for a safe and effective subsequent keratoplasty. We excluded patients with defective eyelid closure.

Evaluation of study outcomes (diagnosis of LSCD, recovery of the corneal epithelium after SLET, maintenance of proper corneal epithelization after keratoplasty) were based on limbal and corneal IVCM findings, preoperatively and at monthly intervals up to 12 months after surgery and, in the case of keratoplasty, for the 12 months thereafter.

We performed slit-lamp biomicroscopy examination to document clinical aspects of the ocular surface. Both examinations were also performed in the fellow eye to describe the limbus, preoperatively and at the excision site following limbal biopsy at 1-month.

Presence of symptoms was evaluated at each visit, asking patients about the occurrence of discomfort due to burning, pain, and photophobia.

### 2.2. In Vivo Confocal Microscopy

We conducted IVCM examinations using the HRT III Rostock Cornea Module diode-laser 670-nm scanning microscope (Heidelberg Engineering GmbH, Heidelberg, Germany) under topical anesthesia with 0.4% oxybuprocaine cloridrate (Benoxinato cloridrato Intes, Alfaintes, Napoli, IT). A drop of 0.2% polyacrylic gel (Lacrinorm, Farmigea, Pisa, Italy) was placed between the contact cap and the objective lens and between the contact cap and the contact lens applied to protect the corneal surface. The patient was asked to fixate on a target-light with the contralateral eye, which was moved to scan the central, temporal, superior, nasal, and inferior sectors. The same operator performed all examinations, obtaining scans using the sequence mode acquiring one image per second. The live color image of the examined eye from a side camera embedded in the instrument allowed real-time confirmation of the location of the scanned area.

We diagnosed LSCD based on the identification of conjunctival phenotype over the corneal surface, where conjunctival basal cells appeared larger than the corneal basal epithelial cells, exhibiting two morphological patterns: cells with prominent nuclei with ill-defined borders and cells with bright borders, dark cytoplasm, and no visible nuclei. Presence of goblet cells and cystic formations were considered further signs of conjunctivalization [16].

Sub-basal nerve plexus and inflammatory cells (dendritic cells, DC) were also investigated, since a decline or complete absence of sub-basal nerves and presence of DC with different patterns has been reported [17,18].

We graded LSCD as a total in the case of the absence of cells with corneal epithelial phenotype and the presence of cells with conjunctival phenotype over the entire scanned corneal surface [1]. Moreover, the absence of the normal architecture of Vogt’s palisade at the limbus (i.e., limbal crypts and focal stromal projections, FSP) was confirmed [18].

We considered surgical success as soon as we found these hallmarks and if maintained until the last follow up, instead the surgery was considered partially successful or failed at 12 months after SLET if either mixed corneal-conjunctival epithelial cells over the cornea and transition zone in one to three quadrants were present (partially successful), or conjunctival cells over the cornea and no epithelial transition zone were detected (failed) [13,15].

### 2.3. Slit-Lamp Biomicroscopy

The ocular surface and adnexa were preoperatively examined to evaluate the corneal status (neovascularization, epithelial defects, fluorescein staining, pannus, and stromal thinning), conjunctiva (inflammation, keratinization), and eyelids (symblepharon, trichiasis and blepharitis).

During the first postoperative month, the biomicroscopy evaluation aimed at monitoring possible adverse reactions mainly related to inflammatory response or the loss of sclerocorneal pieces, maintenance of the bandage contact lens (BCL) over the corneal surface, and the closure of the epithelial defect.

At monthly visits, following SLET and keratoplasty, we evaluated possible recurrence of superficial vascularization and the epithelial integrity by fluorescein staining.

### 2.4. Surgical Technique

SLET was performed following the technique described by Sangwan et al. [2]. Briefly, under topical anesthesia, we excised a portion of 2 × 2 mm limbal tissue up to the clear cornea from the superior limbal area in the unaffected contralateral eye and stored it in balanced saline solution. Peribulbar anesthesia was induced in the eye with LSCD and the corneal surface was exposed by removal of the fibrovascular corneal pannus following a 360° conjunctival peritomy. We grafted human amniotic membrane (AM), epithelial side up, over the cornea, secured this with fibrin glue (TISSEEL, Baxter AG, Vienna, Austria), and trimmed the margins to fit the peripheral conjunctival borders. We divided the limbal sclerocorneal tissue into eight to ten small pieces and fixed these with fibrin glue onto the AM, in a circular fashion, sparing the optical zone. A soft BCL was placed over the transplant and eyelids were closed with strips for 72 h. Oral prednisone 1 mg/kg/die (Deltacortene, Bruno Farmaceutici, Roma, Italy) was administered for three days, then tapered over 15 days, a proton pump inhibitor was given once daily for 15 days, and ciprofloxacin 500 mg bid (Ciproxin, Bayer, Milano, Italy) for five days was administered.

### 2.5. Postoperative Management

Following opening of the eyelids, at the first postoperative visit three days after SLET, patients were examined weekly until a complete corneal epithelial closure was observed. Single-dose netilmicin sulfate 0.3% (Nettacin, SIFI, Catania, Italy) was administered qid and maintained until complete epithelial closure. Dexamethasone sodium diphosphate 0.15% (Etacortilen, SIFI, Catania, Italy) was given six times daily for 45 days, gradually tapered thereafter at monthly intervals, and discontinued based on the clinical condition. Topical preservative-free lubricants (Thealoz gel, Thea Pharmaceuticals, Clermont-Ferrand, France) were administered qid and maintained throughout the study period. Patients maintained BCL for three weeks, and then it was replaced at each visit until a stable epithelial closure lasting for at least two months was observed. After a successful SLET was confirmed, patients were scheduled for keratoplasty to improve visual outcomes. In the case of postoperative complications, these were managed following our standard clinical protocols.

### 2.6. Statistical Analysis

Efficacy and safety analyses are reported by treatment, since patients underwent SLET and keratoplasty in the same eye and each treatment was considered to have a unique safety and efficacy profile. We expressed the results of the descriptive analyses as median and interquartile range (IQR) for quantitative variables as these did not follow a normal distribution according to the Kolmogorov–Smirnov normality test, while counts and percentages were used for categorical variables. SPSS software (IBM SPSS Statistics for Windows, V.22.0, IBM Corp., Armonk, NY, USA) was used to perform all statistical analyses.

## 3. Results

We included 10 patients (nine males) with a median age of 59.5 years (IQR 13 years) (Table 1). All patients were diagnosed with total LSCD, exhibiting upon IVCM evaluation the presence of cells with conjunctival phenotype over the entire scanned corneal surface, with an absence of limbal crypts and focal stromal projections at the sclerocorneal junction over the corneal periphery.

### 3.1. Preoperative Evaluation

All patients reported burning or pain and photophobia in the affected eye and were taking topical lubricants. Four patients additionally used topical combined antibiotic-corticosteroid therapy four times daily. Biomicroscopy examination revealed superficial corneal vessels, conjunctivalization, and absence of Vogt’s palisades and focal stromal projections in all patients. In two patients, severe central corneal thinning was detected, with this region covered by a thin conjunctival layer.

**Figure 1:**
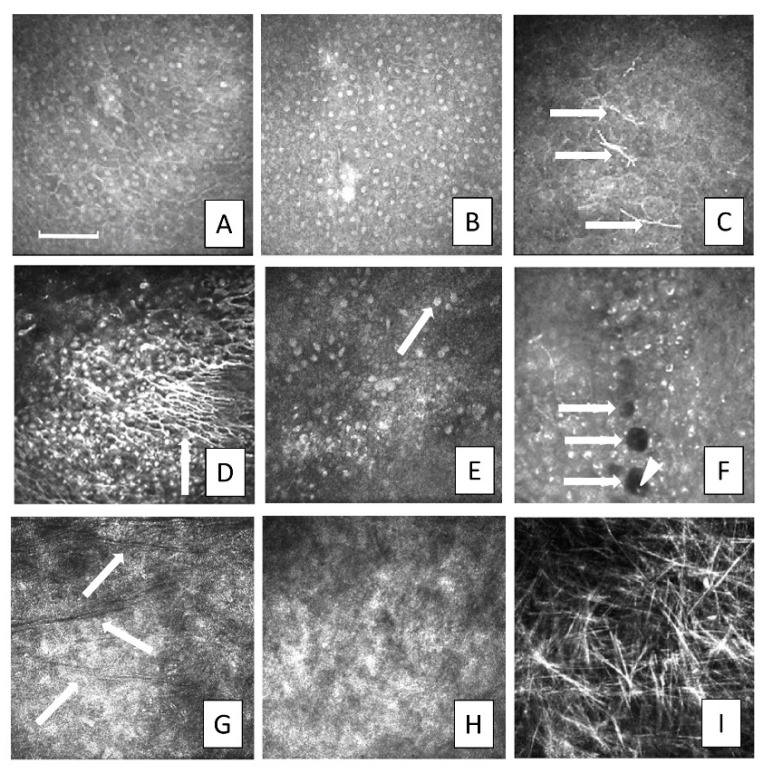
Preoperative In Vivo Confocal Microscopy findings of the corneal surface impaired by limbal stem cell deficit. (**A**) Superficial conjunctival cells populating the corneal surface, hyper-reflective, with bright nuclei and ill-defined borders. (**B**) Basal conjunctival cells with prominent nuclei and ill-defined borders. (**C**) Basal conjunctival cells with bright borders, dark cytoplasm, barely visible nuclei, and Langerhans cells visible among basal cells (arrows). A net of activated dendriform cells (**D**, arrow). (**E**) Goblet cells are hyper-reflective, larger than surrounding epithelial cells, and round to oval in shape (arrow). (**F**) Microcysts (arrows) are specific to conjunctival pannus, and appear as round or oval dark shapes with bright borders; small, white, or grey spherical elements, likely mucin, can sometimes be detected inside the microcysts (arrowhead). (**G**) Bowman’s layer appears as dark folds overlying the anterior stroma (arrows) in patients with a history of keratoplasty. (**H**) Only bare stroma was detectable at that level in the remaining patients. (**I**) Stromal scarring is characterized by copious hyper-reflective needles in the mid-stroma, representing severe keratocyte apoptosis (images are 400 × 400 µm, the bar is 100 µm).

A healthy ocular surface and preserved limbal anatomy were observed in all donor fellow eyes. Upon IVCM examination (Figure 1), in the affected eye, all patients had cells with a conjunctival phenotype on the corneal surface and displayed an absence of an epithelial transition zone. Six patients had cells with prominent nuclei with ill-defined borders, three patients had cells with bright borders, dark cytoplasm and no visible nuclei, while one patient showed both patterns. Goblet cells were found in three patients while six patients had cystic formations. Mature DC were observed in the central cornea at the level of basal epithelium in four patients and in none of the corneas were clear sub-basal nerves detected. Bowman’s layer was clearly identifiable only in patients who underwent previous keratoplasty after the injury. In the other patients, anterior corneal layers were so altered because of the injury that the Bowman’s layer was indistinguishable. The absence of a normal limbal architecture was confirmed in all cases.

### 3.2. Postoperative Evaluation

The main safety and efficacy outcomes after SLET and after keratoplasty are summarized in Table 2.

At the last follow up, the best-corrected visual acuity (BCVA) increased by a median of two lines (IQR = 2 lines) after SLET and by a median of six lines (IQR = 1) for patients who underwent subsequent keratoplasty.

**Figure 2:**
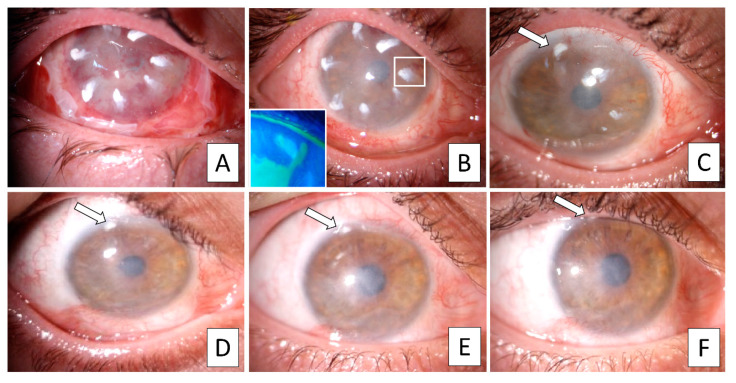
Longitudinal slit lamp biomicroscopy indicating the progressive disappearance of limbal biopsy fragments after Simple Limbal Epithelial Transplantation (SLET). (**A**) At one week after SLET, the amniotic membrane (AM) and the limbal biopsy fragments are evident; (**B**) after one month, the AM is completely resorbed and the limbal biopsy fragments present more indistinct margins; the epithelial growth from the limbal biopsy fragment is distinguishable as unstained epithelium in the context of stained AM (cobalt blue light after fluorescein staining, bottom left); (**C**) at 2-months, few reduced-sized fragments are still visible; (**D**–**F**) from 3, 6, and 12 months, respectively, no limbal biopsy fragments were detectable on the clear cornea. Notably a fragment on the peripheral supero-temporal area persisted (**F**, arrows), likely following its integration within the limbo-scleral tissue (representative results from patient no. 10).

Biomicroscopy evaluation after surgery showed the limbal biopsy fragments on the AM, with the AM itself completely resorbed by the end of the first month, while the biopsy fragments persisted over the clear cornea (Figure 2).

In patient no. 7, examination one month after SLET showed no AM or limbal biopsy fragments on the corneal surface. Regeneration of the corneal epithelium was not obtained throughout the study period, and conjunctival epithelium was detected in the corneal periphery while a bare central cornea persisted. This patient was considered as a case of early SLET failure.

**Figure 3:**
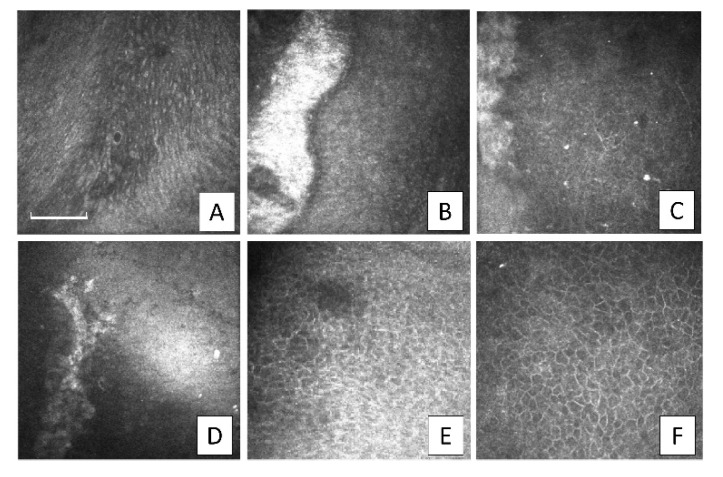
In Vivo Confocal Microscopy (IVCM) details of the evolution of a transplanted sclerocorneal piece after Simple Limbal Epithelial Transplantation (SLET) in the same patient of Figure 2. (**A**) Preoperative appearance of the basal conjunctivalized cells lying on the cornea. (**B**) Small hyper-reflective limbal biopsy fragment at 1-month postoperative, surrounded by small cells with scarcely distinguishable borders. (**C**) At 2-months, the same limbal fragment appeared attenuated with jagged margins. (**D**) At 3-months, it was barely visible. (**E**) At 6-months, the limbal fragment completely disappeared and clear corneal epithelium was detected. (**F**) At 12-months, the result was maintained (representative results from patient no. 10; images are 400 × 400 µm, the bar is 100 µm).

Upon IVCM examination in the other patients, small islets of epithelial cells with amniotic characteristics, interspersed among cells of corneal epithelial phenotype, were observed at 1-month postoperative but these were no longer visible from the 2-month follow-up examination. The AM stroma was also distinguishable at the 2- and 3-month follow-up examinations. The limbal biopsy fragments were visible, surrounded by small cells with scarcely distinguishable borders. The biopsy fragments exhibited jagged margins at the 2-month follow-up and were progressively undetectable from the 3-month (Figure 3).

**Figure 4:**
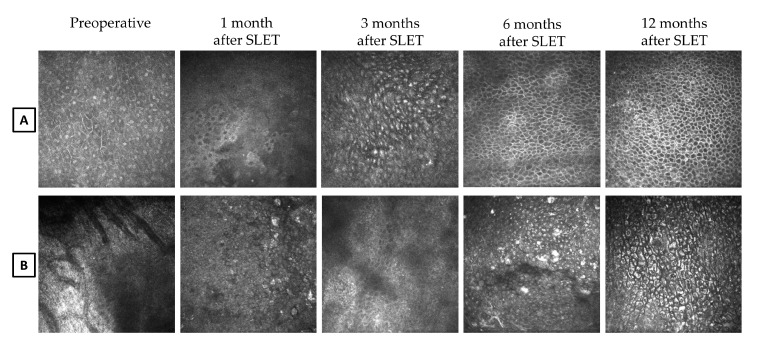
Basal epithelium in the central cornea in a successful (**A**) and in a partial successful (**B**) patient, preoperatively and at 1, 3, 6, and 12 months after Simple Limbal Epithelial Transplantation (SLET). Preoperative, in (**A**) is clearly distinguishable a conjunctivalized epithelium and in (**B**) the presence of neovascularization. After SLET, in the successful case (**A**) we could distinguish at 1-month, remnants of the amniotic membrane epithelium with well-defined bright thick borders and homogeneous cytoplasm lacking nuclear details;at 3 months, immature epithelium with small indistinct cell borders, bright nuclei, and a high nucleus-to-cytoplasm ratio; at 6- and 12-months, a normal basal corneal epithelium; neither goblet cells nor cystic formations were detectable in all corneal sectors. After SLET, in the partial successful case (**B**) we could distinguishat 1- and 3-months, epithelial cells scarcely identifiable with small cell body, poorly defined bright borders, dark cytoplasm, and no visible nuclei; at 6-months, the detectable epithelial cells were enlarged, hyper-reflective, and had activated nuclei with a decreased nucleus-to-cytoplasm ratio;at 12-months, the corneal surface was intact, and cells maintained a dysmorphic pattern (representative results from patient no. 1, (**A**), and patient no. 3, (**B**); images are 400 × 400 µm, the bar is 100 µm).

Starting from the 3-month follow-up after SLET, the renewing corneal epithelial cells appeared with indistinct borders, bright nuclei, and a high nucleus-to-cytoplasm ratio (Figure 4A). Thereafter, we observed an intact corneal epithelium represented by cells exhibiting a normal corneal phenotype. At the 5-month postoperative evaluation after SLET, a multi-layered epithelium with corneal phenotype was detected in the central cornea in eight out of 10 patients.

**Figure 5:**
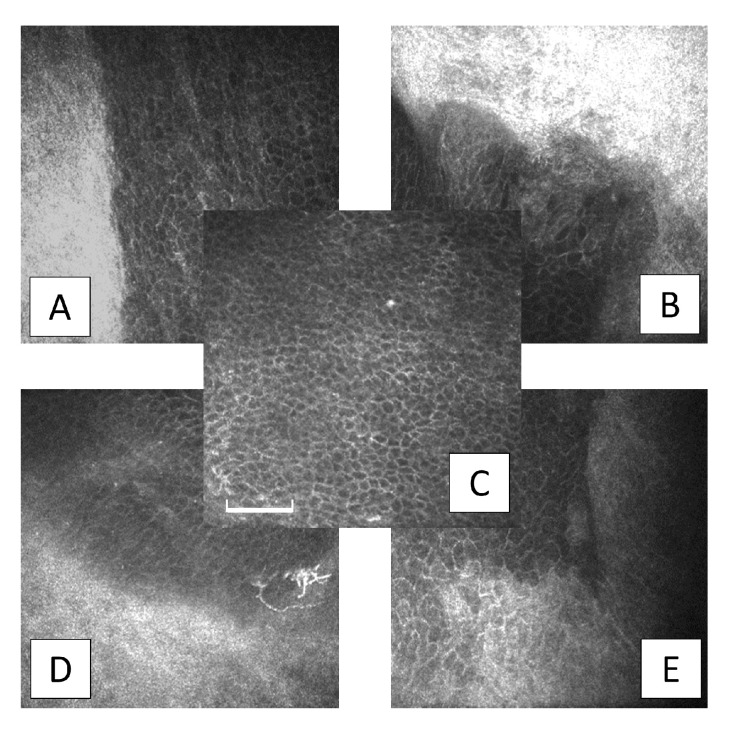
Central epithelium and transition zone at 6-month evaluation after Simple Limbal Epithelial Transplantation (SLET). (**A**,**B**,**D**,**E**) distinct epithelial transition zone in all four peripheral corneal sectors; corneal cells are well-defined, darker, and smaller than their conjunctival counterpart; (**C**) regular and mature basal corneal epithelium in the central cornea. (C denotes central, A temporal, B superior, D inferior, E nasal corneal sectors; Images are 400 × 400 µm, the bar is 100 µm).

A clearly distinguishable peripheral corneal-conjunctival epithelial transition zone was observed over the four corneal quadrants in seven patients within six months after SLET, and in one patient at the 9-month evaluation (Figure 5).

In patient no. 3, a complete surface re-epithelialization was observed seven months after surgery and a transition zone was detected only in two corneal sectors at the 11-month follow-up. This patient was judged as a partial success (Figure 4B).

We documented an increase in DC density compared to baseline at the first month, particularly in the central cornea, likely due to resident peripheral DC stimulated to migrate toward the central cornea, differentiating into a mature and activated phenotype, as previously reported [19]. An improvement in symptoms was noted in all successful patients.

### 3.3. Surgical Interventions after Simple Limbal Epithelial Transplantation (SLET)

Patients no. 5 and no. 6 underwent AM transplantation three months after SLET because of a positive Seidel test in the central cornea. These patients underwent penetrating keratoplasty (PK) three months later, following the confirmed maintenance of the epithelial transition zone in all sectors.

Twelve months post-PK, all patients maintained a clear and intact corneal epithelium by biomicroscopy and IVCM examinations, showing a well-defined transition zone in all the peripheral corneal sectors. Three more patients underwent keratoplasty eight to 12 months after SLET, after the detection of normal corneal epithelium over the corneal surface and a restoration of the peripheral transition zone; two of these patients underwent deep anterior lamellar keratoplasty (DALK) and one underwent PK.

All grafts were 7.5–8 mm in diameter to minimize the risk of involving part of the transition zone, and interrupted sutures were used in all cases, with the knots buried at the donor side.

**Figure 6:**
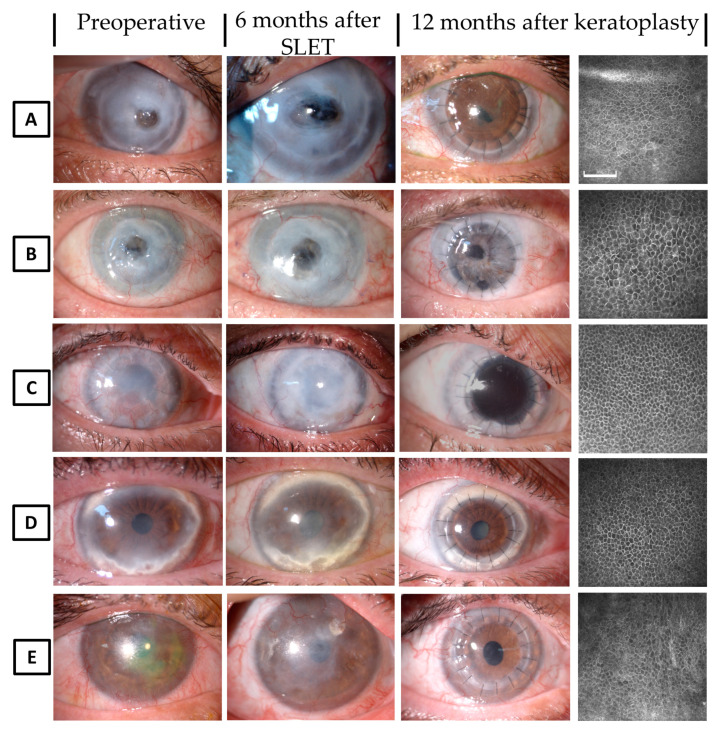
Clinical course of Simple Limbal Epithelial Transplantation (SLET) and subsequent keratoplasty after corneal surface reconstruction. Corneal appearance in patient no. 5 (**A**), 6 (**B**), 1 (**C**), 2 (**D**), and 10 (**E**) before SLET, at 6-months after SLET and 12-months after keratoplasty. In Vivo Confocal Microscopy (IVCM) evaluation of the central basal epithelium 12 months after keratoplasty (right side of the rows) showed a normal corneal phenotype in all cases. Clear grafts, with no evidence of epithelial failure, were observed up to 1-year after keratoplasty (IVCM images are 400 × 400 µm, the bar is 100 µm).

At the 12-month follow-up after DALK or PK, all transplanted corneas exhibited a clear and intact corneal epithelium and epithelial transition zone in the peripheral corneal quadrants (Figure 6).

### 3.4. Adverse Events and Complications

No adverse events or complications occurred in the donor fellow eyes after limbal donor tissue excision. Patients no. 5 and no. 6 had a positive Seidel test in the central cornea at the 3-month evaluation after SLET. Increased intraocular pressure was found in patient no. 4, three months after SLET, as a response to topical corticosteroid therapy. An additional preservative-free timolol 0.05%/dorzolamide 0.02% combination was administered twice daily and was maintained until the discontinuation of the corticosteroid therapy. The failed SLET (patient no. 7) reported worsening of itching, discomfort, and photophobia. We reviewed this patient weekly and protection with BCL, preservative-free topical lubricants, and antibiotics were prescribed.

## 4. Discussion

The renewal of a phenotypically normal corneal epithelium onto the corneal surface can be recognized and followed-up after SLET using IVCM, based on the recognition of cell morphology, corneal tissue modifications, and healing processes. In our experience, IVCM examinations have been safely repeated starting from the first month postoperatively, when the AM was almost completely integrated and the grafted limbal fragments were firmly adherent to the underlying stroma.

IVCM was useful for both diagnosis of LSCD and in judging the efficacy of SLET. This result was further confirmed by the maintenance of a clear corneal epithelium 12 months after keratoplasty, as indirect evidence of the maintenance of the limbal stem cell function in the host peripheral cornea.

In agreement with previous reports, the different epithelial patterns described in our case series of LSCD diagnosis by IVCM confirmed that cells with prominent nuclei with ill-defined borders were the prevalent feature in the conjunctival pannus [20]. An additional feature of conjunctival epithelium that has been described is the presence of goblet cells and intraepithelial cysts [21]. Unlike conjunctival epithelial cells, goblet cells have a limited role in the diagnosis of LSCD and a 20–50% detection rate in the cornea is reported [1,17,20]. In fact, in our series, only a few patients showed goblet cells supporting the diagnosis for LSCD. Moreover, preoperatively, we found various degrees of stromal alterations including a highly reflective acellular stromal matrix related to the modification or degeneration of collagen structure due to the original cause of LSCD [22], and hyper-reflective needles, likely secondary to apoptosis of keratocytes and altered innervation that may lead to stromal collagen breakdown and degeneration.

Our clinical success rate was similar to that obtained in other studies: Basu et al. published long term results of SLET of 125 eyes. The clinical success was maintained in 76% of the eyes at 12 months [23]. Gupta et al. reported a similar success rate (70%) in 30 eyes [24].

In our series, an improvement in the visual acuity was present after SLET and was even more marked after keratoplasty. The visual outcome is essential for the functional rehabilitation of these patients; nonetheless, a lack of vision improvement does not necessarily indicate a failure because SLET restores the limbal epithelial stem cells (LESCs) function but does not treat the residual corneal stromal opacity. The improvement of symptoms in such patients often could itself be considered as a success for the patient with an improvement in quality of life, besides the visual function [6].

In the failed case, there was an early disappearance of the AM and limbal fragments within the first month. This is reported in the literature as the major risk factor associated with SLET failure [25]. Amescua et al. proposed a modification of the original technique with the apposition of a second AM over the limbal fragments and sutured to the peripheral conjunctiva, this could reduce the risk of early loss of limbal fragments and further support their survival in the immediate postoperative period [26].

The features of the epithelial cells detected soon after SLET were comparable to that of corneal epithelial cells replicating and migrating during the healing processes after epithelial debridement [27]. Subsequent observations during the follow-up showed a normal corneal epithelium.

The main aim of the present study was to gather evidence, for the first time, for the assumption that the detection of epithelium with a corneal phenotype and an epithelial transition zone by IVCM is sufficient to continue on the therapeutic path with a follow-on corneal keratoplasty. Epithelium with a corneal phenotype and a peripheral epithelial transition zone was found in the majority of our patients within six months after SLET. Five patients underwent keratoplasty soon following these observations and results were maintained 12 months after keratoplasty, proving the worthiness of our hypothesis. Anyway, as for other techniques of stem cell transplantation, limbal crypts or palisade-like structures were not detectable in our patients. An experimental study demonstrated the presence of the renewal capability of cells retrieved from the sclerocorneal site previously harvested for limbal autograft transplantation, although limbal sections from four out of six patients showed that the typical anatomic features of the palisades of Vogt were lost [28]. We can therefore speculate that a new stem cell reservoir site could develop after SLET, but its exact location and structure remain unclear. For these reasons, the presence of the transition zone by IVCM could be used as a marker of the restored limbal stem cell function. In patients that underwent AM transplantation 3-months after SLET because of a Seidel positive test, the subsequent resolution of the corneal epithelial gap in the central cornea with proper corneal epithelium is a further confirmation of the reliability of the transition zone as a marker for the outcomes, that may be more relevant than the clinical findings.

Additionally, IVCM enabled the detection of inflammatory cells in the corneal tissue, presenting us with the opportunity to visualize the inflammatory status of the cornea [29]. We did not consider the inflammatory response observed in our patients as a sign of impending risk for graft failure, but mainly as a response to surgery. Both the presence of AM, which provides an anti-inflammatory effect, and the ongoing topical corticosteroid therapy, allowed a rapid suppression of the inflammation that was maintained in the follow-up of successful patients.

Our study has several limitations. An immunohistochemical examination of the cells present in the excised corneas after SLET would aid in characterizing the still unclear phenomenon of cell renewal after this kind of transplantation, while a larger sample size would lend additional power to the results and observations obtained. Even if the real utility of IVCM in such cases is to provide qualitative information crucial for subsequent clinical decision-making, a quantitative evaluation of IVCM parameters would better standardize the interpretation of these results and their unambiguous implementation. Finally, a longer follow-up period would indicate whether the results obtained here will persist in the longer term, over several years.

A recent meta-analysis on outcomes of limbal stem cell transplantation has shown that only 26% of the studies considered used diagnostic tests such as IVCM to confirm the diagnosis of LSCD, recognizing this as a limitation [6]. We believe that the lack of such examinations is even more limiting in the interpretation of clinical outcomes. In conclusion, IVCM is useful to diagnose LSCD and primarily allows for the characterization of the healing process at the corneal surface after SLET, supporting clinical and surgical decisions for the complete rehabilitation of these challenging patients.

## Figures and Tables

**Table 1:** Demographic characteristics and clinical history of patients.

No.	Age (Years)	Sex	Cause of LSCD (Year)	Previous Surgery (Year)	Ocular Surface and Corneal Appearance
1	61	F	Severe bacterial keratitis (1979)	PK (2015–2017)	Fibrovascular pannus with irregular surface
2	68	M	Alkali burn (1978)	EDTA (2017), AMT (2017)	Complete conjunctivalization, stromal haze, peripheral lipid degeneration
3	59	M	Thermal injury (1999)	PHACO, IOL, PK (2015)	Fibrovascular pannus, irregular surface and recurrent PEDs
4	58	M	Alkali burn (2016)	-	Fibrovascular pannus with superficial stromal opacification
5	49	M	Alkali burn (2012)	PK (2016) CLET (2014)	Fibrovascular pannus, complete corneal opacification with central thinning
6	73	M	Severe fungal keratitis (2011)	PK (2011)	Fibrovascular pannus, complete corneal opacification with central thinning
7	60	M	Alkali burn (2016)	AMT (2016)	Conjunctivalization with superficial vessels and recurrent PEDs
8	34	M	Alkali burn (2006)	-	Irregular surface with inferior keratinization and superficial vessels
9	52	M	Alkali burn (2015)	CLET (2017)	Fibrovascular pannus with stromal haze
10	61	M	Alkali burn (1974)	-	Conjunctivalization with superficial vessels and recurrent PEDs

AMT, amniotic membrane transplantation; CLET, cultivated limbal epithelial transplantation; EDTA, Ethylenediaminetetraacetic acid; F, female; IOL, intraocular lens; M, male; PED, persistent epithelial defect; PHACO, cataract phacoemulsification; PK, penetrating keratoplasty.

**Table 2:** Outcomes and complications after simple limbal epithelial transplantation (SLET) and keratoplasty.

	After SLET				After Keratoplasty	
No.	Detection of Transition Zone, Month	Complications	BCVA Improvement Compared to Baseline, Lines	Subsequent Surgeries	BCVA Improvement Compared to Baseline, Lines	Follow-up from SLET/Keratoplast, Months
1	3	-	2	PK	6	8/12
2	9	-	1	DALK	5	10/12
3	11	-	2	-	-	24/-
4	4	Increased IOP	3	-	-	22/-
5	2	Seidel positive test in the central cornea	0	AMT, PK	7	6/12
6	3	Seidel positive test in the central cornea	0	AMT, PK	6	6/12
7	not obtained	AM early reabsorbed, PED, worsening of discomfort	-	-	-	12/-
8	3	-	2	-	-	22/-
9	2	-	3	-	-	18/-
10	6	-	1	DALK	6	11/12

AM, amniotic membrane; AMT, amniotic membrane transplantation; DALK, deep anterior lamellar keratoplasty; EDTA, ethylenediaminetetraacetic acid; IOP, intraocular pressure; PED, persistent epithelial defect; PK, penetrating keratoplasty; BCVA best corrected visual acuity.
